# Structural Diversity of Nickel and Manganese Chloride Complexes with Pyridin-2-One

**DOI:** 10.3390/molecules25040846

**Published:** 2020-02-14

**Authors:** Saša Petriček

**Affiliations:** Faculty of Chemistry and Chemical Technology, University of Ljubljana, Večna pot 113, 1000 Ljubljana, Slovenia; sasa.petricek@fkkt.uni-lj.si; Tel.: +386-1-479-85-12

**Keywords:** Ni(II) complexes, Mn(II) complexes, pyridin-2-one, O-bridging ligand, crystal structure, thermal stability

## Abstract

Reactions of NiCl_2_·6H_2_O and pyridin-2-one (C_5_H_5_NO = Hhp) afforded novel molecular complexes, i.e., mononuclear [NiCl_2_(Hhp)_4_] (**1**), dinuclear [NiCl_2_(Hhp)(H_2_O)_2_]_2_^.^2Hhp (**3**) and [Ni_2_Cl_4_(Hhp)_5_]·2MeCN (**4**), and an ionic complex [Ni(Hhp)_6_]Cl_2_ (**2**). Single-crystal X-ray analyses revealed two modes of Hhp ligation in these complexes: a monodentate coordination of carbonyl oxygen in all of them and an additional *µ*_2_-oxygen bridging coordination in the dinuclear complex **4**. Three bridging molecules of Hhp span two nickel(II) ions in **4** with a 2.9802 (**5**) Å separation of the metal ions. Complex **3** is a chlorido-bridged nickel dimer with a planar Ni_2_(*µ*-Cl)_2_ framework. Hydrogen bonds and parallel stacking arrangements of the Hhp molecules govern the connectivity patterns in the crystals, resulting in 1D structures in **1** and **5** or 2D in **3**. A single manganese compound [MnCl_2_(Hhp)_4_] (**5**), isostructural to **1**, was isolated under the similar conditions. This is in contrast to four nickel(II) chloride complexes with Hhp. Thermal analyses proved the stability of complexes **1** and **3** in argon up to 145 °C and 100 °C, respectively. The decomposition of **1** and **3** yielded nickel in argon and nickel(II) oxide in air at 800 °C.

## 1. Introduction

**Scheme 1:**
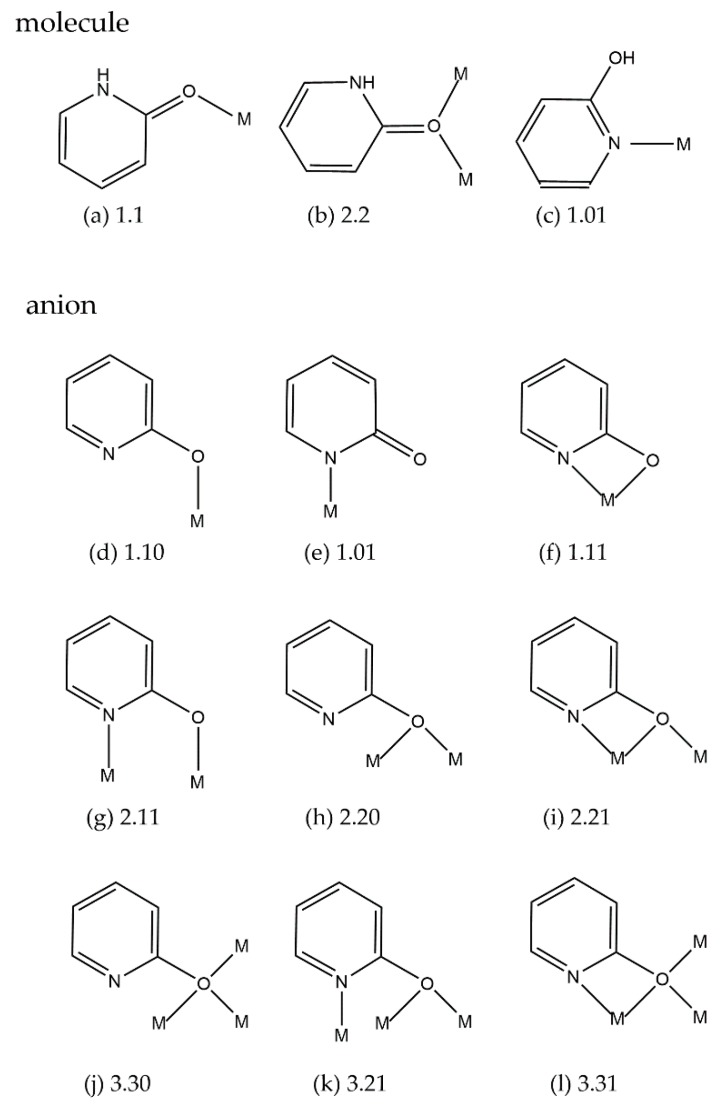
Coordination modes of (**a**,**b**) Hhp; (**c**) 2-hydroxypyridin; (**d**–**l**) hp^−^ with Harris notation [1].

Pyridin-2-one (Hhp), its anionic form (hp^−^) and the chloro (Hchp, chp^−^) or methyl (Hmhp, mhp^−^) derivatives of these two species are bound as ligands in many transition-metal compounds. The diverse coordination modes of these ligands, the stabilization of complexes by hydrogen bonds and other weak intermolecular interactions result in various structural motifs for a plethora of compounds with interesting magnetic, catalytic, and biochemical properties. In contrast to the three binding modes of the neutral Hhp, nine ligation possibilities were described in the literature for hp^−^ and derivatives, providing a huge structural diversity among the complexes of these ligands (Scheme 1).

The anion hp^−^ and its derivatives can act as “carboxylate-like” dinucleating triatomic bridging ligands (Scheme 1g). This *N*,*O* bridging coordination plays an important role in the long-term research of metal-metal multiple bonds, starting with the isolation of dinuclear mhp^−^ complexes of Group-6 metal(II) ions [2]. The influence of hp^−^ derivatives on the M–M bond lengths (M = Cr, Mo, W, Ru, Rh) in [ML_4_] (L = mhp^−^, chp^−^, fhp^−^ (6-fluoropyridin-2-olate)) was widely studied [3,4,5,6].

The similar nature of pyridonates and carboxylates was clearly demonstrated in dinuclear copper compounds displaying distinctive paddle-wheel structures. The hp^−^ as *N*,*O* bridging ligands can fully or only partly replace the methanoate bridges in complexes of [Cu_2_(*µ*-hp)_4_(Hhp)_2_] and [Cu_2_(*µ*-HCO_2_)_2_(*µ*-hp)_2_(Hhp)_2_] [7].

The first example of the mhp^−^ anion as a link between hetero metal ions in the 2.11 fashion was found in [MoW(mhp)_4_]·CH_2_Cl_2_ at the end of the 1970s [8]. The extensive research of heterometallic complexes with hp^−^ anions was later focused on the improved syntheses of superconductors. The trinucleating nature of hp^−^ with the 3.21 ligation yielded copper lanthanoide (Y, La, Nd, Gd or Dy) complexes in which metal ions are linked into heterometallic assemblies. These compounds were considered as the precursors in formation of high-temperature superconductors [9,10,11,12]. The coordination spheres of the lanthanum and dysprosium ions in these bimetallic clusters are fulfilled by Hhp molecules coordinated as terminal O-donor ligands [9,11]. A similar type of compound with the same 3.21 bonding fashion of hp^−^ prepared later was targeting possible single molecule magnet (SMM) behavior. A weak SMM behavior was detected for gadolinium, terbium, and dysprosium compounds with copper and hp^−^ [13]. A single ion magnet behavior was also observed in two bimetallic (Co(III), Ln(III) = Tb, Dy) complexes with Schiff base ligands and the terminal O-donor ligation of chp^−^ to lanthanide ions [14].

No manganese or nickel complexes with Hhp and its molecular or anionic derivatives were characterized at the time of an excellent review by Rawson et al. in 1995 [15]. Since then, a few manganese and many nickel polynuclear complexes with hp^−^, chp^−^ and mhp^−^ achieving various coordination modes have been synthesized in the search for compounds with special magnetic properties. Anions chp^−^ or mhp^−^ and additional bridging ligands—carboxylates or triethanolamine—connect the manganese ions to chains or polynuclear cages. A chelating *N*,*O* plus a *µ*_2_-oxygen bridging ligation of chp^−^ (Scheme 1i) was observed in a 1D manganese acetate complex [16], while merely a monodentate coordination of chp^−^ or mhp^−^ via an oxygen atom is found in a hepta- or in a nonanuclear manganese cage [17,18]. Only the oxygen atoms of chp^−^ ligated in the 3.30 fashion also participate in the bonding of manganese ions in an icosanuclear complex, which is a mixed-valent cluster consisting of four Mn(II) and 16 Mn(III) ions [19]. Almost a whole spectrum of ligation modes is adopted by hp^−^ and its derivatives in nickel complexes. These ligands were used to control the aggregation of metal ions into clusters. The formation of insoluble nickel phosphonate coordination polymers was prevented by the coordination of hp^−^ derivatives achieving more soluble clusters instead of extended lattices. The four bonding modes, i.e., 2.20, 2.21, 3.21, and/or 3.31 were adopted by chp^−^ in five complexes of deca- or dodecanickel phosphonates with chp^−^ and pivalate [20]. Anions mhp^−^ act as mono-, di- or trinucleating ligands displaying four coordination types in a single compound of a decanuclear nickel complex with pivalate (Scheme 1f,i,k,l) [21]. In contrast to the diverse binding fashion of these ligands in each of the previously cited complexes, exclusively one type of coordination is involved in the others. Just *µ*_2_-oxygen atoms derived from two or three chp^−^ anions bridge the nickel ions in a decanuclear acetate cluster [22] or in a linear trinuclear complex, respectively [23]. A single coordination mode of the chp^−^ and mhp^−^ anions—the 2.21 fashion—is also assumed in some hepta- or undeca-nickel pyridonates [24,25]. A curious feature of some nickel complexes is the chelate coordination of three chp^−^ in [Ni(chp_3_]^2−^, a counterion to cationic polynuclear nickel clusters [26,27]. The chelate coordination of hp^−^ is accomplished in a mononuclear nickel complex with a N_3_-macrocycle [28]. Interestingly, the 1,3-bridging coordination of hp^−^ or its derivatives has not been adopted by manganese or nickel complexes, although complexes of some other 3*d* metals—copper or chromium—include this type of bonding (2–4,7).

In contrast to hp^−^ anions—stabilizing high-nuclearity metal cages—are the Hph, Hchp, or Hmhp complexes, mostly mono- or di-nuclear species, if no additional bridging ligands are included. The first Hhp complexes with metal(II) (Mn, Co, Ni, Cu, Zn and Cd) perchlorates, tetrafluoroborates and nitrates were characterized by means of spectroscopy, elemental analyses, magnetic measurements and X-ray powder-diffraction analyses in the early 1970s [29,30]. While exclusively homoleptic ionic species [M(Hhp)_6_]^2+^ were proposed in the perchlorates and tetrafluoroborates, the nitrates were either solely molecular [M(Hhp)_4_(NO_3_)_2_] (M = Mn, Cu, Zn, Cd) or molecular and ionic (M = Co, Ni). Some Hhp complexes of manganese(II), cobalt(II), nickel(II), or copper(II) chloride were also prepared many years ago. The formula MCl_2_·Hhp was suggested for all four metals, but just two of them enabled the isolation of the additional adducts CoCl_2_·3Hhp and CuCl_2_·2Hhp [31]. The terminal ligation of Hhp molecules via a carbonyl oxygen atom in homoleptic [M(Hhp)_6_]^n+^ (M = Fe(III), Co(II), Cu(II) [32,33,34] or in heteroleptic ion [Co(Hhp)_4_(H_2_O)_2_]^2+^ [35] was later confirmed by single-crystal X-ray analyses. The substitution of large perchlorate in [Cu(Hhp)_6_](ClO_4_)_2_ [34] by chloride does not only modify the homoleptic complex ion to heteroleptic species, but also reduces the coordination number of the copper. Various Hhp copper chloride complexes were reported, a mononuclear [CuCl_2_(Hhp)_3_] and two dinuclear, [CuCl(*µ*_2_-Cl)(Hhp)]_2_, [CuCl_2_(Hhp)(*µ*_2_-Hhp)]_2_ [36]. The crystallographic characterization of the above mentioned Hhp nickel(II) or manganese(II) complexes has not been accomplished, only four structures of Hhp derivatives [M(L)_4_X_2_] (L = Hchp, Hmhp, X = Cl, NCS) were reported for both metal ions [37,38].

The Hhp molecules are coordinated as terminal, auxiliary ligands via carboyl oxygen in complexes of many 3*d* metals. In this way, they fulfill the coordination spheres of vanadium in acetylacetonate [39], manganese in alkoxide [40], iron [41,42] or copper in acetates [43], and zinc in phosphonate complexes [44]. A rare reversible single-crystal to single-crystal transformation was observed in a trinuclear iron(III) acetate. The coordination of each iron site is completed by an O-donor atom derived either from Hhp or water molecule. A weakly bonded water molecule can be replaced by methanol in a reversible process [41]. Manganese and copper complexes were studied because of their interesting magnetic properties. A copper complex with four bridging pyrazine and two terminal Hhp acts as an extremely well-isolated 2D-antiferromagnetic layer [45]. Magnetic interactions were also studied in two manganese complexes, a 1D compound of azido bridged Mn(II) [46] or dinuclear bis-*µ*-alkoxo Mn(III) [40], both of them include Hhp as a terminal, ancillary ligand.

The nitrogen atom in 2-hydroxypyridine is mostly coordinated as a donor atom to the more polarizable 4*d* and 5*d* metal ions (Ru, Ir, Pt) [47,48,49,50,51,52]. Platinum complexes were widely studied to elucidate interactions of *cis*-[PtCl_2_(NH_3_)_2_] and nucleic acid bases or their analogues [47]. This type of coordination was detected in a complex of the first-row transition-metal—in a tetranuclear cobalt complex—only few years ago [53]. The tetranuclear complex of cobalt has been of interest as a catalyst in an oxygen-evolution reaction [53].

Carbonyl oxygen in Hhp can also act as dinucleating ligand in the *µ*_2_-bridging fashion. Two Hhp molecules connect two copper ions in dinuclear [Cu_2_(Hhp)_2_(*µ*-Hhp)_2_X_4_] (X = Cl, Br, tfa = trifluoroacetate) [36,54]. A Hhp molecule bridges two silver(I) ions via exocyclic oxygen atom in a molecular [Ag_4_(Hhp)_2_(*µ*-Hhp)_2_(*µ*_2_-tfa)_4_] [55]. In contrast to the prevailing Hhp complexes of low nuclearity, the *µ*-oxygen bridging coordination of Hhp to silver(I) ions creates chains in an ionic compound [Ag(Hhp)_2_(*µ*-Hhp)_2_]_n_(PF_6_)_n_ [56]. Ag(I) ions are in an octahedral environment of four bridging and two terminal Hhp molecules. Hmhp molecules ligated in the 2.2 fashion contribute to the design of hexanuclear nickel cages in some nickel benzoato and pivalato complexes [57,58]. The same ligation of Hmhp was found in a dodecanuclear nickel-phosphonate cluster [59].

We have continued our study of the first-row transition-metal chloride complexes with hydroxypyridines [60]. The coordination of Hhp to nickel(II) or manganese(II) ions was the focus of the research, since no structural data of Hhp nickel compounds and only two manganese complexes including Hhp as an auxiliary ligand were found in the Cambridge Structural Database (CSD, Version 5.40) [40,46,61]. Reactions of nickel(II) or manganese(II) chloride hydrates and Hhp have been investigated in details. Our goal was to prepare diverse complexes of these two metals by varying the conditions, particularly the molar ratio of the reactants and solvents used in the syntheses. We were interested in the influence of the selected metal ion on the structural diversity of the Hhp complexes. The results are compared to the structural variety of the analogous copper halide complexes. The impact of ancillary ligands and solvate molecules on the supramolecular aggregations is discussed. The thermal decomposition of the complexes was evaluated and correlated with the structural parameters.

## 2. Results and Discussion

### 2.1. Synthetic Aspects

**Scheme 2:**
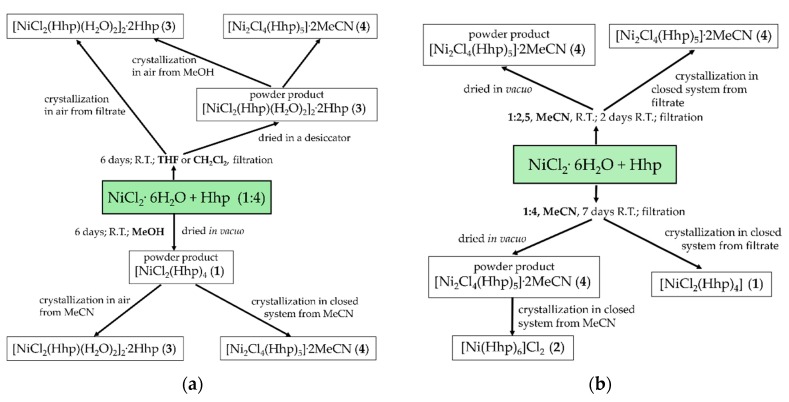
Reactions of NiCl_2_^.^6H_2_O and Hhp (**a**) in methanol, THF, or dichloromethane, (**b**) in acetonitrile.

The products afforded by the reactions of NiCl_2_^.^6H_2_O and Hhp strongly depend on the solvent and the molar ratio of the reactants (Scheme 2). A powder product of complex [NiCl_2_(Hhp)_4_] (**1**) is achieved in methanol, [NiCl_2_(Hhp)(H_2_O)_2_]_2_·2Hhp (**3**) in tetrahydrofuran, dichloromethane, or acetone, and [Ni_2_Cl_4_(Hhp)_5_]·2MeCN (**4**) in acetonitrile. The formations of powder products **1** and **3** were proven by X-ray powder-diffraction measurements that correspond well to X-ray powder-diffraction patterns calculated from crystal structures. The formation of powder product **4** was confirmed by an IR spectrum. Powder product **1** was formed when a solution obtained in a reaction of NiCl_2_·6H_2_O and Hhp (molar ratio 1:4) in methanol was dried in vacuo. Reactions of NiCl_2_·6H_2_O and Hhp in tetrahydrofuran or acetonitrile resulted in suspensions, which were first left to settle and then filtrated. The precipitate obtained in acetonitrile was dried in vacuo yielding complex **4** and the precipitate in tetrahydrofuran dried in a desiccator, resulting in complex **3**. Crystallization of powder product **1** or **3** from acetonitrile yields crystals **3** in the presence of moisture or crystals **4** in a closed water-free system. Crystals [Ni(Hhp)_6_]Cl_2_ (**2**) were grown from a solution of powder product **4** in acetonitrile in an aqua-free environment. The reaction time and the molar ratio of the reactants in acetonitrile control the isolation of the crystals **1** or **4** from filtrates in closed water-free systems (Scheme 2b). Crystals **1** were obtained from the filtrate with the higher, and **4** from the filtrate with the lower, molar ratio of nickel ions to Hhp.

Complex [NiCl_2_(Hhp)_4_] (**5**) was obtained only in a two-step reaction. In the first step the reaction of MnCl_2_·2H_2_O and chlorotrimethylsilane in THF resulted in [MnCl_2_(THF)_x_] as confirmed by an IR spectrum. Chlorotrimethylsilane was used to remove all the water in the coordination sphere of manganese(II) ions similar to the syntheses of manganese(II) complexes with diglyme (di(2-methoxyethyl) ether) or H_2_dhp (3-hydroxypyridin-2-one) [60,62]. Ligated molecules of THF were substituted by molecules of Hhp in the following step achieving complex **5**. In spite of altering the solvents and the molar ratio of manganese(II) ions to Hhp, only complex **5** was isolated in all the experiments. 

Only compound **3** is stable in air, while a slow decay was observed for complex **1** and fast decays for complexes **2**, **4,** and **5** in the presence of water.

### 2.2. IR Spectra

Broad bands around 3000 cm^−1^ in all the spectra indicate the presence of N–H groups that are engaged in hydrogen bonds. Only in the spectrum of complex **3** with coordinated water molecules are broad bands visible above 3300 cm^−1^ because of the O–H(water) stretching vibrations. Similarly, as N–H are O–H bands observed at low values (3426 and 3357 cm^−1^) due to the strong hydrogen bonding of the ligated water molecules to chloride ions and the solvate molecules of Hhp in **3**. C=O vibrations characteristic of Hhp are shifted in the spectra of all complexes due to the Hhp coordination to metal ions via carbonyl oxygen atom and hydrogen bonds [29,38]. Shifts of the C=O stretching vibrations from 1681, 1636, and 1606 cm^−1^ in solid Hhp to lower wave numbers were observed in all the complexes. In complexes **1** and **4** (1642, 1596, 1586 cm^−1^ in **1** and 1640, 1599 cm^−1^ in **4**) the shifts of the C=O stretching vibrations are more pronounced than in complex **3** with the coordinated and solvate molecules of Hhp (1652, 1614, 1587 cm^−1^). IR spectra of the isostructural complexes **1** and **5** are identical.

### 2.3. Description of the Structures

Crystals of the nickel complexes—**1**, **2**, and **4**—suitable for X-ray analyses, were obtained in closed systems by the slow evaporation of solvents under reduced pressure at room temperature over a period of a few days. Only crystals of the stable complex **3** were isolated from the solution in an open beaker. The structural variety of the nickel and copper halide complexes with Hhp is comparable despite the different coordination numbers of the central metal ions [36]. The ligands render a distorted octahedral environment around the nickel(II) ions in all the complexes **1**–**4**, while copper displays lower coordination numbers in halide complexes, four or five. The nickel complexes are mono- (**1**) or dinuclear molecules (**3**,**4**) and cations in the homoleptic specie (**2**). Hhp coordinates via the carbonyl oxygen atom as the terminal atom in all complexes, an additional bridging coordination of the ligand was found only in **4**. Complex **4** is a unique example of triply-bridged nickel ions in a dinuclear compound connected exclusively by neutral O-donor ligands. In [Cu(Hhp)_2_X_2_]_2_ (X = Cl, Br, tfa), only two Hhp molecules link the copper ions [36,54]. Crystals of the single manganese complex, **5**, were achieved in the same way as crystals **1**, **2** and **4**. In spite of the various solvents and the molar ratios of the manganese(II) ions to Hhp applied in the reactions, only a molecular complex, **5**—isostructural to **1**—was gained. A summary of the crystal data collections and refinement parameters for **1**–**5** are listed in Table 1.

CCDC—1973332 (**1**), 1973334 (**2**), 1973336 (**3**), 1973335 (**4**), and 1973333 (**5**) contain the supplementary crystallographic data for this paper. These data can be obtained free of charge at http://www.ccdc.cam.ac.uk/conts/retrieving.html, or from the Cambridge Crystallographic Data Centre, 12 Union Road, Cambridge, CB2 1EZ, UK; Fax: +44-1223-336033; or e-mail: deposit@ccdc.cam.ac.uk.

#### 2.3.1. Crystal Structure of **1**, [NiCl_2_(Hhp)_4_], **2**, [Ni(Hhp)_6_]Cl_2_ and **5**, [MnCl_2_(Hhp)_4_]

**Figure 1:**
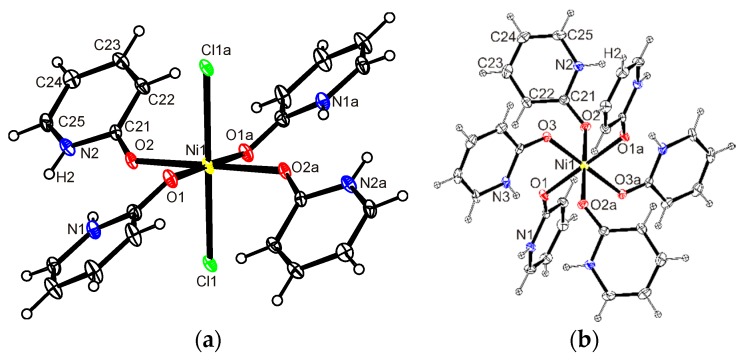
ORTEP drawing of (**a**) [NiCl_2_(Hhp)_4_] (**1**) and (**b**) [Ni(Hhp)_6_]Cl_2_ (**2**). Atoms are represented by displacement ellipsoids at the 25% probability level. Hydrogen atoms are shown as spheres of arbitrary radii.

**Figure 2:**
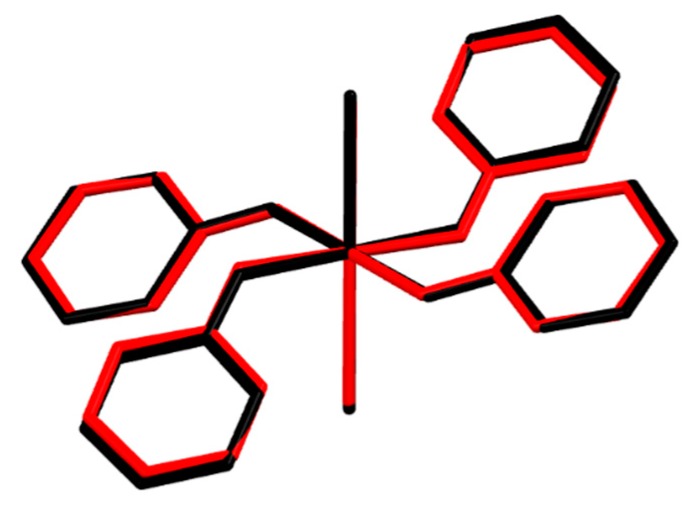
Structure overlay of [NiCl_2_(Hhp)_4_] (**1**) (red) and [MnCl_2_(Hhp)_4_] (**5**) (black). For the sake of clarity, hydrogen atoms are omitted.

The Hhp molecules—four in **1** or **5** and six in **2**—are coordinated to metal ions via carbonyl oxygen in the monodentate manner (Figure 1). O_6_ or Cl_2_O_4_ donor sets in these three complexes render octahedral environments around the central ions. The coordination sphere of central ion in **1** or **5** is fulfilled by two chloride ions in the *trans* positions. The distortion from the regular octahedral geometry is small in all the compounds (Table 2). A half of these complex species is included in the asymmetric unit with metal ions located in the inversion center. Complex **5** is isostructural to complex **1** which is confirmed by selected geometric parameters (Table 1 and Table 2) and by the overlay of both structures depicted in Figure 2.

The metal-to-donor atom distances are comparable in complexes **1** and **5** with respect to the differences in the manganese(II) and nickel(II) ion radii [63]. In complex **5,** the manganese-to-donor atom distances are in the same range as in the complex of 3-hydroxypyridin-2-one [MnCl_2_(H_2_dhp)_4_] [60]; a shorter and a longer Mn–O distance is found in both of them. However, in **1** and **5**, the M–O bonds are shorter and the M–Cl bonds are longer than the corresponding bonds in the similar [MCl_2_(Hchp)_4_] (Ni–O 2.103(2) Å, Mn–O 2.204(3) Å, Ni–Cl 2.361(1) Å, Mn–Cl 2.506(1) Å) [38].

The average Ni–O distance is shorter in the ionic complex **2** (2.047(2) Å) than in the molecular **1** (2.057(4) Å). Relatively uniform M–O distances, as in **2**, were found for the homoleptic ions [Co(Hhp)_6_]^2+^ (from 2.0695(15) Å to 2.1014(14) Å) [33], while the Cu–O distances in similar ions range from 2.00(1) Å to 2.29(1) Å or even 1.922(2) Å to 2.546(2) Å due to the significant influence of the Jahn–Teller effect [34,54]. Interestingly, an analogue [Ni(H_2_dhp)_6_]^2+^ was not detected in a series of nickel chloride complexes with 3-hydroxypyridin-2-one [60].

Short C–O bonds in **1**, **2** and **5** confirm the coordination via carbonyl oxygen in accordance with the many similar [M(L)_4_X_2_], with L in the formula standing for Hhp, Hchp, or Hmhp [35,38,64,65]. Only in three of the [M(L)_4_X_2_] complexes (M = Mn, Co, Ni) is the coordination of Hmhp subscribed to the hydroxyl oxygen atom due to the positions of the hydrogen atoms found from the differences in the Fourier synthesis. The reported ligation seems uncertain, because shorter C–O distances (1.264(3)–1.279(3) Å) also indicate double bonds in these [M(Hmhp)_4_(SCN)_2_] complexes and a coordination of carbonyl instead of hydroxyl oxygen [37]. A decrease of the nitrogen atom basicity by methyl in the 6-position and the predomination of the enol form are also unexpected [15].

**Figure 3:**
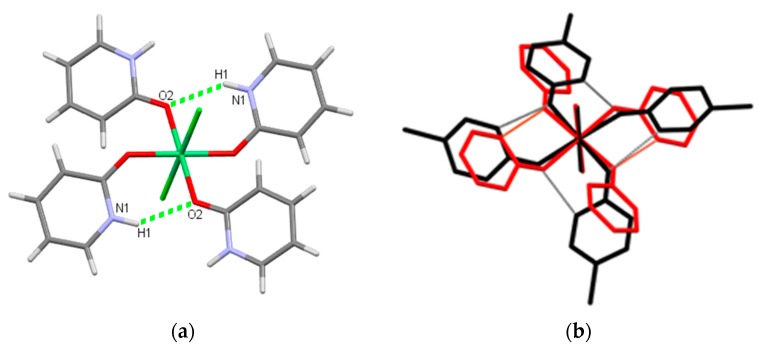
(**a**) Intramolecular hydrogen bonds in **1** and **5**. (**b**) The structure overlay of [NiCl_2_(Hhp)_4_] (**1**) (red) and [NiCl_2_(Hchp)_4_] (black) [38]. For the sake of clarity, hydrogen atoms in (**b**) are omitted.

Four ligated molecules are connected by two intramolecular hydrogen bonds N1–H1⋯ O2 in two pairs in **1** or **5** (Figure 3a, Table S1). In contrast, all four organic ligands are bonded to a quasimacrocyclic metal complex through hydrogen bonding (Figure 3b, the black structure) in many other reported compounds [M(L)_4_X_2_]^n+^ (*n* = 0 or 2, M = Mn, Co, Ni, Cu; L = Hhp, Hchp, Hmhp; X = H_2_O, Cl^−^, NO_3_^−^, SCN^−^) [35,37,38,64,65]. This remarkable variation in the intramolecular hydrogen bonding could be explained by the orientation of the organic ligands. The nitrogen atoms in pairs of symmetrically independent ligands in **1** or **5** are directed towards each other in contrast to the nitrogen atoms in other reported [M(L)_4_X_2_] [35,37,38,64,65]. The two planes through symmetrically independent aromatic ligands also bent far away from the equatorial plane, defined by four oxygen atoms (36.69°, 44.14° in **1** and 37.08(7)°, 42.63(6)° in **5**). This is in contrast to a lesser shift of planes through Hhp in [Co(Hhp)_4_(H_2_O)_2_]^2+^ (5.92°, 11.88°) [35] or Hchp in [NiCl_2_(Hchp)_4_] (18.43°) [38]. A deviation of the ligand planes from the equatorial plane was clearly proven to influence the packing in two [Cu(Hchp)_4_(H_2_O)_2_](CLO_4_)_2_ compounds and resulted in different characteristics for these polymorphs [65].

**Figure 4:**
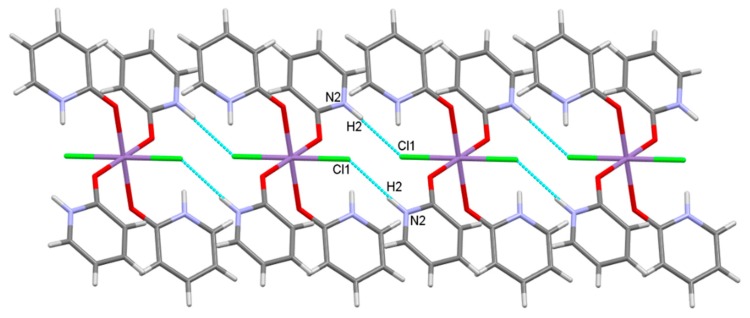
Intermolecular hydrogen bonds in a section of a chain in **1** or **5**.

[MCl_2_(Hhp)_4_] molecules in **1** and **5** are linked by intermolecular hydrogen bonds into chains parallel to the *a* axis (Figure 4). N–H groups, not involved in intramolecular hydrogen bonds, are donors and chloride ions in the adjacent molecules are acceptors of intermolecular hydrogen bonds resulting in a graph-set motif *R*_2_^2^(12) [66,67].

**Figure 5:**
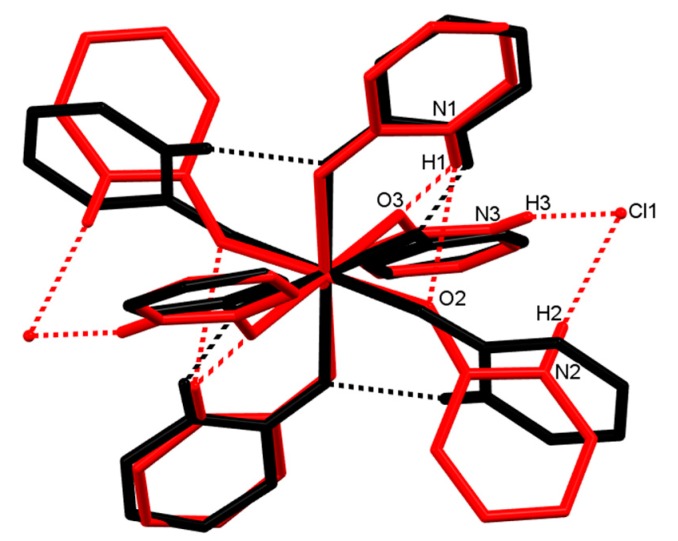
Structure overlay of [Ni(Hhp)_6_]^2+^ (**2**) (red) and [Co(Hhp)_6_]^2+^ (black) [33]. For the sake of clarity, only hydrogen atoms involved in hydrogen bonds are depicted.

In complex **2,** all the donors of hydrogen bonds, N–H groups, participate in intra- and inter-molecular hydrogen bonds (Figure 5, the red structure). The N1–H1 groups in the two ligated Hhp are donors of bifurcated intramolecular hydrogen bonds to acceptors, four carbonyl oxygen atoms of neighboring ligands. Thus, two groups composed of three ligated molecules linked by intramolecular hydrogen bonds are generated in a cation. The carbonyl oxygen atoms in two Hhp molecules are not involved in hydrogen bonds. Two groups of interlinked ligated Hhp are also found in the homoleptic cobalt(II) complex, although the connectivity mode differs (Figure 5, the black structure). Four N–H groups in [Co(Hhp)_6_]^2+^—not only two as in **2**—are engaged as donors of linear (2-center) intramolecular hydrogen bonds [33]. In contrast to the nickel and cobalt complexes, they are all six ligands bonded by intramolecular hydrogen bonds into a macrocycle in [Cu(Hhp)_6_](ClO_4_)_2_] [34]. Two N–H groups are donors of bifurcated and two of linear intramolecular hydrogen bonds in the copper compound. This diversity in the intramolecular hydrogen bonds in [M(Hhp)_6_]^2+^ can be explained by the orientations of the aromatic ligands (Figure 5) shown by the M–O–C angles and the angles between the planes determined by the ligated molecules. The M–O–C angles are in range from 127.8(1)° to 128.6(1)° for M = Ni, from 127.7(1)° to 132.3(1)° for M = Co [33] and from 105.9(1)° to 132.2(2)° for M = Cu [34]. The angles between the planes defined by the ligands are also significantly different in the [M(Hhp)_6_]^2+^ cations. These angles are similar and low if M = Co (31.3°, 34.0°, 39.6°) [33] and more heterogeneous in the nickel(II) (29.1°, 57.1°, 85.7°) and copper(II) (55.8°, 65.9°, 86.9°) analogues [34]. 

The intermolecular hydrogen bonds in **2** connect only a cation and two adjacent anions (Figure 5). The four protonated ring nitrogen atoms in **2**, which are not engaged in the intramolecular hydrogen bonding, are donors and two chloride ions are acceptors, each acting as an acceptor of two hydrogen bonds. Additional weak interactions occur between the Hhp molecules coordinated in adjacent cations, linking cations into chains parallel to the *a* axis. An aromatic *sp*^2^-CH group is a hydrogen donor (soft acid) and a hetero aromatic ring (soft base) plays the role of a CH-acceptor in a T-shaped C–H⋯ π interaction. The relevant parameters are H24⋯ centroid distance 2.89 Å, C24–H24⋯ Cg angle 152° and C24⋯ centroid distance 3.7334 Å [68]. An alike simple association of a cation to two anions via intermolecular hydrogen bonds was also observed in the homoleptic copper complex with Hhp [34], while the aggregation of [Co(Hhp)_6_]^2+^ and CoCl_4_^2−^ into chains was found in the cobalt complex [33].

#### 2.3.2. Crystal Structure of **3**, [NiCl_2_(Hhp)(H_2_O)_2_]_2_ 2Hhp

**Figure 6:**
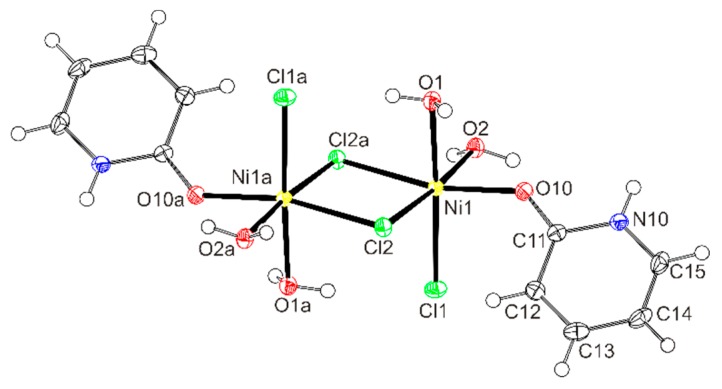
ORTEP drawing of complex molecule [NiCl_2_(Hhp)(H_2_O)_2_]_2_ in **3**. Atoms are represented by displacement ellipsoids at the 25% probability level. Hydrogen atoms are shown as spheres of arbitrary radii.

A pair of six-coordinated nickel ions is connected by two bridging chlorides in the centrosymmetric dinuclear complex **3** (Figure 6). A half of the complex molecule is included into the asymmetric unit with the inversion center in the middle between two nickel ions. Three chloride ions, a terminal and two bridging, and three oxygen atoms, one originated in Hhp, two in water, are coordinated to each nickel ion in **3** in the *fac* octahedral mode. Two water molecules are in the *cis* positions. A minor distortion of the octahedron is obvious from the relevant bonding parameters listed in Table 3.

Interestingly the Ni–Cl distances to both—a bridging and a terminal—chloride ions in **3** are almost the same. More often, a shorter bond to a terminal and a longer one to a bridging chloride are determined, as in [NiCl_2_(diglyme)]_2_ (2.3443(5) Å, 2.4447(5) Å) [62]. The shortest Ni–O bond in **3** is the one to the carbonyl oxygen and a significant variation of the two Ni–O(water) distances is worth mentioning. The average Ni–Cl distance in **3** (2.403(2) Å) is a slightly longer and the Ni–O distance (2.081(4) Å) is shorter than the corresponding distances in [NiCl_2_(diglyme)]_2_ (2.378(2) Å), 2.104(4) Å) [62].

A planar arrangement of two nickel ions connected by two bridging chlorides (Ni_2_(*µ*-Cl)_2_ framework) was found in many dinuclear complexes with Cl_2+a_N_b_O_c_ (a + b + c = 4; a = 0 or 1; b = 0–4, c = 0–3) donor sets [62,69,70,71,72,73,74,75,76]. In contrast, a similar Cl_3_O_3_ environment of nickel as in **3** according to CSD was found only in two complexes with organic O-donor molecules, diglyme, or carbacylamidophosphates [62,69]. The only *mer* isomer among these three complexes was isolated for [NiCl_2_(diglyme)]_2_ [62]. The separation of nickel ions in **3** is similar to the nickel carbacylamidophosphate (3.419 Å) [69] and a bit shorter than in [NiCl_2_(diglyme)]_2_ (3.522 Å) [62] or in a bipyridine complex (3.441 Å) with a Cl_2_N_2_O_2_ donor set in a coordination sphere [70]. Shorter separations (3.338–3.371 Å) of two nickel ions connected by two bridging chloride ions were found in some complexes with Cl_3_N_2_O donor sets, which were utilized in ethylene polymerization [71,72,73]. By increasing the number of N-donor atoms to three or four (Cl_3_N_3_ or Cl_2_N_4_ donor sets) in the surroundings of the nickel ions in dinuclear complexes the distances between adjacent nickel ions increase to over 3.65 Å [74,75,76]. Shorter distances between the metal ions are accompanied by more acute Ni–Cl–Ni angles. As the Ni⋯ Ni distances range from 3.338 Å to 3.683 Å in the mentioned complexes the Ni–Cl–Ni angles vary from 87.99° to 95.22 3° [71,75,76]. The Ni–Cl–Ni angle in **3** is in accord with these data. The plane of coordinated Hhp and the (Ni_2_(*µ*-Cl)_2_ plane in **3** form an angle of 41.22(7)°. However, the planes of aromatic ligands and the Ni_2_(*µ*-Cl)_2_ framework are either nearly coplanar and/or perpendicular in the majority of dinuclear nickel(II) chloride complexes with this type of framework [70,71,72,74].

**Figure 7:**
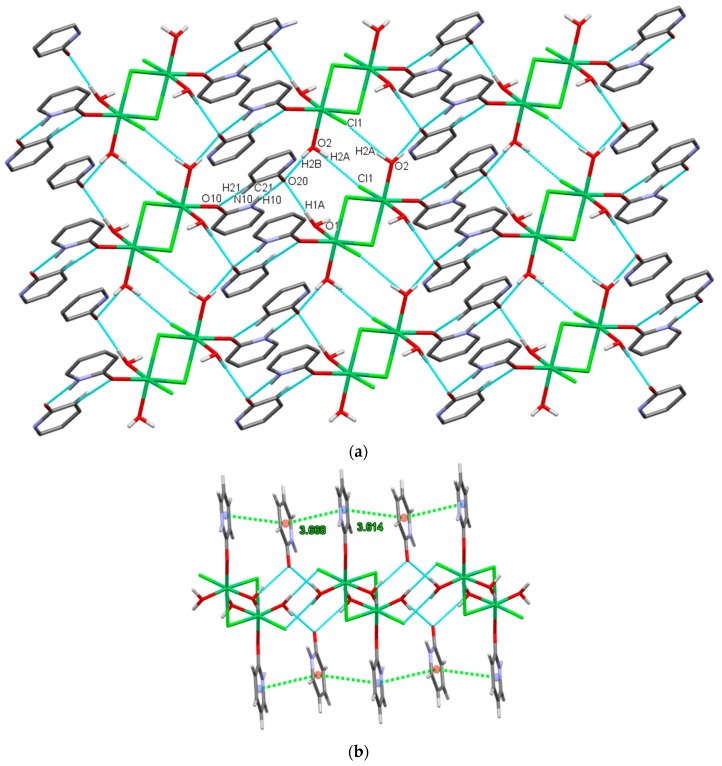
Complex and solvate molecules in **3** are connected to layers parallel to the *ac* plane by (**a**) hydrogen bonds and (**b**) an off-center parallel stacking arrangement of Hhp presented by centroid–centroid distance (green dashed lines).

Complex **3** displays intermolecular hydrogen bonds generating layers parallel to the *ac* plane (Figure 7, Table S2). Each complex molecule [NiCl_2_(Hhp)(H_2_O)_2_]_2_ forms altogether twelve hydrogen bonds with six closer Hhp solvate molecules and two neighboring complex molecules. Complex molecules participate in building chains parallel to the *a* axis through the O2–H2A⋯ Cl1 hydrogen bonds (graph-set motif *C*_2_^2^(10)[*R*_2_^2^(8)]). Complex and solvate molecules are bound via O1–H1A⋯ O20 and N10–H10⋯ O20 (graph-set motif *C*_4_^2^(18)[*R*_4_^2^(16)]) into chains parallel to the *c* axis [66,67]. Solvate molecules are acceptors and donors of intermolecular hydrogen bonds contributing significantly to the formation of planes by linking chains parallel to the *a* and *c* axes. A carbonyl oxygen O20 in a Hhp solvate molecule is an acceptor of three D–H⋯ O20 hydrogen bonds (D = O or N). Each solvate Hhp molecule also connects to a ligated one by N10–H10⋯ O20 and C21–H21⋯ O10 hydrogen bonds into a dimeric unit, which also enhances the stability of the solvent molecules in compound **3**.

The close proximity of coordinated and solvate Hhp molecules within a layer allows an off-center parallel stacking arrangement in **3** (Figure 7b) [77]. The relevant parameters of these alternating interactions in **3** are: centroid–centroid distances 3.614(1) or Å 3.668(1) Å, interplanar distances 3.252(1) Å or 3.336(1) Å, a dihedral angle 6.67°, and offset angles 22.6° or 27.6° [78]. 

#### 2.3.3. Crystal Structure of **4**, [Ni_2_Cl_4_(Hhp)_5_]·2MeCN

**Figure 8:**
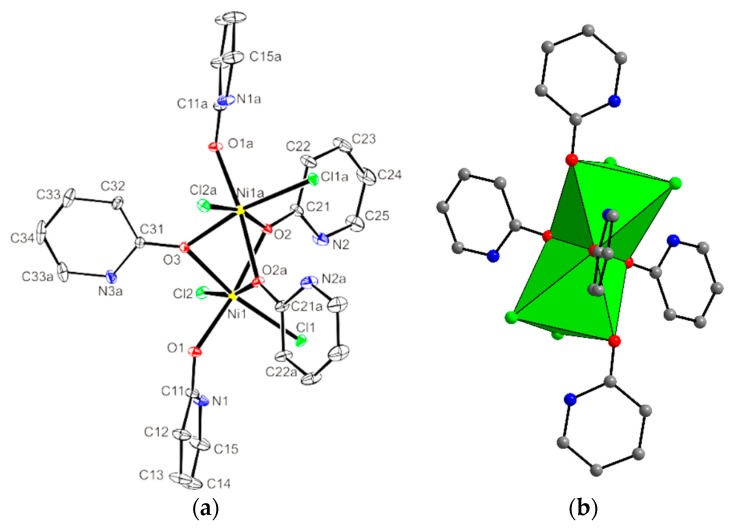
Drawing of complex molecule [Ni_2_Cl_4_(Hhp)_5_] in **4**. (**a**) Atoms are represented by displacement ellipsoids at the 25% probability level. (**b**) Two face-sharing octahedra. For the sake of clarity, hydrogen atoms are omitted.

The two nickel ions in **4** are triply bridged by carbonyl oxygen atoms derived from Hhp molecules (Figure 8). The two chloride ions in *cis* positions and a terminally coordinated Hhp molecule complete the octahedral environment of each metal ion. A significant distortion of the octahedron is obvious from the relevant bonding parameters listed in Table 4. Two space groups were considered in solving and refining structure **4**, *C*2/*c* (no. 15) was chosen over *Cc* (no. 9) due to the better results obtained in the refinement. This selection imposes a two-fold rotation axis through the bridging O3=C31 carbonyl group. As a consequence, the asymmetric unit includes half of a complex molecule. According to the symmetry requirements, only half of the atom positions in the bridging Hhp ligated via O3 are independently determined; therefore, at one site a 50% occupancy by N3 and 50% by C32 was proposed.

A comparison reveals shorter Ni–Cl(terminal) bond lengths in **4** than in **1** or **3**. A shorter and two longer Ni–O bonds in **4** are to the terminal and bridging ligands, respectively.

A *µ*_2_-oxygen ligation of Hhp is well documented in the dinuclear copper(II) halides or acetate [36,54] and silver(I) complexes [55,56]. In nickel compounds, only Hhp derivatives–Hchp or Hmhp–were observed as neutral organic O-donor bridging ligands, which in cooperation with other bridging anions connects metal ions in hexa—[57,58], nona—[27] and dodecanuclear [20,59] clusters. Copper ions in dinuclear compounds are linked by two Hhp molecules [36,54], while three molecules are involved in a bridging coordination to a pair of 3*d* metal ions only in a vanadium complex of Hmhp [79]. Three *µ*-oxygen atoms from Hmhp connect the metal ions in a dinuclear V_2_(*µ*-O)_3_ framework resulting in the formation of two face-sharing coordination octahedra. In contrast to three symmetrically bridging oxygen atoms in nickel complex **4**, two distinct bridging ligand types are present in [V_2_Cl_2_(*µ*-(Hmhp)_3_)O_2_], a symmetrical one (V–O 2.0882(2) Å, 2.104(2)Å) and an unsymmetrical one (V–O 2.043(2) Å, 2.345(2) Å) [79]. In a linear trinuclear nickel complex triple oxygen bridges also connect two pairs of neighboring metal ions, but the bridging O-donor ligands are in [Ni_3_(chp)_6_(EtOH)_6_] chp^−^ anions [23]. The distances between two nickel ions in the centers of the face-sharing octahedra in this trinuclear complex are shorter (2.825 Å) than in **4**. Equivalent face-sharing octahedra to those in **4** are found in some dinuclear nickel complexes with a Ni_2_(*µ*-O)_3_ framework. Three bridging oxygen atoms in dinuclear nickel compounds in most cases originate from two phenolate ions and one water molecule [80,81,82,83,84,85,86]. A symmetrical coordination of water and an asymmetrical ligation of phenolate oxygen atoms are found in all cited compounds except one [80]. The separation of nickel(II) ions (from 2.971 Å to 2.988 Å) [80,86] is in the same range, as in **4** or shorter (from 2.857 Å to 2.891 Å) [81,82,83,84,85]. In **4,** there are Ni1–O–Ni1 angles close to 91°, but in the dinuclear complexes with shorter Ni⋯ Ni distances there were three acute angles calculated. In the complexes with a similar separation of nickel(II) ions as in **4** only the Ni–O(water)–Ni angle is acute (less than 82°) and the other two are larger than a right angle (~92°) [80,86].

**Figure 9:**
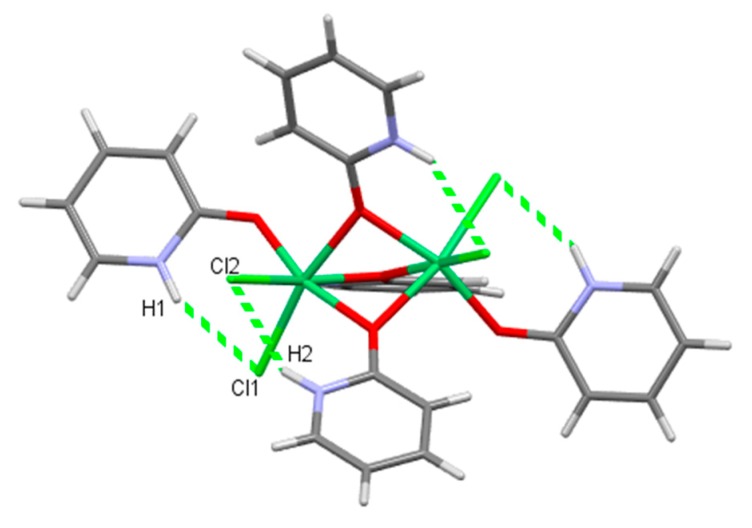
Ligands in **4** are linked by intramolecular hydrogen bonds.

The hydrogen bonding in **4** is entirely intramolecular (Figure 9, Table S3). Four coordinated chloride ions are acceptors of N–H⋯ Cl hydrogen bonds. A comparable hydrogen bonding was found in the dinuclear nickel complexes with a Ni_2_(*µ*-O)_3_ framework, intramolecular hydrogen bonds or the linking of the complex and solvate molecules with no further connectivity prevailing [81,82,83,84,85,86]. Only in a few dinuclear nickel complexes with a similar face-sharing octahedra as in **4** were extended 2D or 3D structures found due to the intermolecular hydrogen bonds enabled by hydroxyl groups at the ends of long side chains in the ligands [80].

### 2.4. Thermal Analysis of ***1*** and ***3***

**Figure 10:**
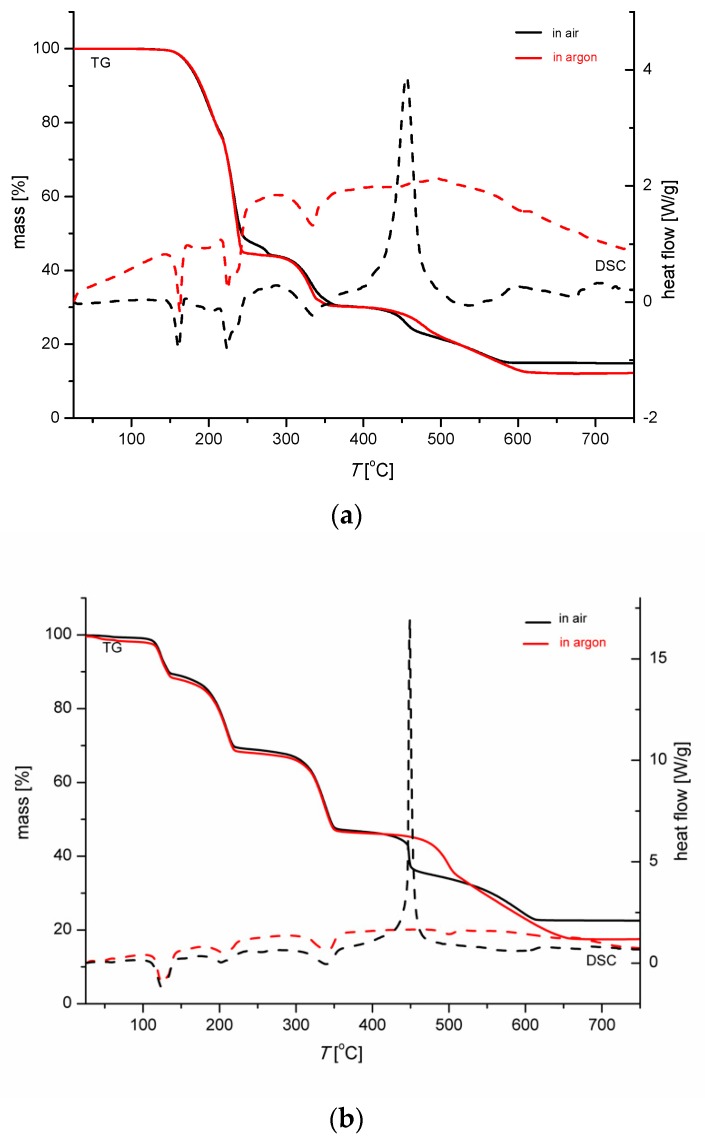
Thermogravimetric (TG—solid line) and differential scanning calorimetry (DSC—broken line) curves for (**a**) **1** and (**b**) **3**.

The results of thermogravimetric (TG) and differential scanning calorimetry (DSC) measurements of complex **1** in argon and in air are shown in Figure 10a. The decomposition of complex **1** in an inert atmosphere begins at a similar temperature as the degradation of a large crystal of Hhp, at 145 °C [87]. TG curve of complex **1** measured in argon shows three distinctive steps. The first step spans the 145 °C–245 °C temperature range with a mass loss of 55.2%, agreeing well with the liberation of three Hhp molecules per formula unit (calculated 55.9%). The following two steps are in the 290–360 °C and 420–610 °C intervals. The total mass loss in all processes, 87.9%, is consistent with the theoretical mass loss of 88.5 for residual nickel. The processes in air and in argon are similar up to 360 °C, while an exothermic reaction follows in air at higher temperature. The peak at 450 °C in the DSC curve indicates the oxidation. The decomposition in air is completed at 580 °C with a total mass loss of 85.4%. This decrease in mass is in a perfect agreement with the calculated one (85.4%) for the formation of NiO. 

The results of the thermogravimetric (TG) and differential scanning calorimetry (DSC) measurements of complex **3** in argon and in air are shown in Figure 10b. Four well-resolved regions of a major decrease in mass were detected in the TG curve measured in argon. The lower thermal stability of complex **3** than **1** was shown by a lower temperature at which the decomposition commences, i.e., 100 °C. The first step continues up to 140 °C. The accompanying mass loss fits well with the release of water molecules that are weakly bonded in the complex according to the longer Ni–O(H_2_O) bonds (Table 3). These results also prove the stabilization of the solvate Hhp molecules in the structure. The solvate molecules are involved in multiple hydrogen bonds and in an off-center parallel stacking arrangement of ligated and solvate Hhp molecules (Figure 7) and released at only a higher temperature. The following three steps in argon are in the 155–200 °C, 275–350 °C, and 460–660 °C temperature ranges. The total mass loss in all processes, 87.9%, is consistent with the theoretical mass loss of 88.5% for residual nickel, similar to the thermal analysis of **1** in argon. A remarkable similarity in the behavior of **3** upon heating in air or in argon was observed up to 400 °C, but at higher temperatures the differences are significant. The intense peak in the DSC curve of **3** in air at 450 °C is at the same temperature as in the DSC curve of **1**, also suggesting an exothermic reaction with oxygen to produce the NiO. The total mass loss during the heating of complex **3** in air up to 800° is 77.7%, slightly less than calculated for the formation of NiO (79%).

## 3. Materials and Methods

### 3.1. General 

Nickel(II) chloride hexahydrate (Merck (Darmstadt, Germany), 98%), manganese(II) chloride tetrahydrate (Merck, 99%), pyridin-2-one (Fluka (Munich, Germany) 97%), chlorotrimethylsilane (Aldrich (St. Louis, MO, USA), 97.0%), and all solvents (tetrahydrofuran, dichloromethane, acetonitrile, methanol, acetone) were used as purchased without any further purification.

The IR spectra (4000–600 cm^−1^) of the samples were recorded using a PerkinElmer Spectrum 100, equipped with a Specac Golden Gate Diamond ATR as a solid sample support. 

Elemental (C, H and N) analyses were carried out with a PerkinElmer 2400 Series II CHNS/O micro analyzer at the University of Ljubljana (Department of Organic Chemistry). 

X-ray powder diffraction data were collected using a PANalytical X’Pert PRO MPD diffractometer with Cu*K*_α1_ radiation (wavelength 1.540596 Å) in the 2θ range from 5° to 70°.

Thermal analyses were performed on a Mettler Toledo TG/DSC 1 instrument (Mettler Toledo, Schwerzenbach, Switzerland) in argon or in air at a 100 mL/min gas flow. Masses of the samples were in the range 7.6–9 mg. Samples in platinum crucibles were heated from room temperature to 800 °C with a heating rate of 10 °C /min. In each case, the baseline was subtracted.

### 3.2. Preparation Procedures

#### 3.2.1. Synthesis of [NiCl_2_(Hhp)_4_], **1**

Solvent (methanol, 20 mL) was added to Hhp (0.560 g, 5.89 mmol) and NiCl_2_**^.^**6H_2_O (0.350 g, 1.47 mmol). The green solution was stirred—for seven days at room temperature or two days at room temperature and additionally an hour at 50 °C—and then dried in vacuo. An amount of 0.727 g of green complex **1** (97.0% yield) was gained. *Anal*. Calc. mass fractions of elements, *w*/%, for C_20_H_20_Cl_2_N_4_NiO_4_ (*M*_r_ = 510.0 g/mol): C, 47.10; H, 3.95; N, 10.99. Found: C, 47.02; H, 4.00; N, 10.93. IR (ATR cm^−1^) 3221 w, 3079 w, 3047 w, 3013 w, 2942 m, 2805 w, 1642 s (ν(CO)), 1597 s (ν(CO)), 1586 vs (ν(CO)), 1541 m, 1460 w, 1418 m, 1402 s, 1361 w, 1266 w, 1255 w, 1219 m, 1150 m, 1105 w, 1085 w, 1016 w, 992 m, 960 w, 912 m, 853 m, 832 m, 793 m, 785 s, 771 vs, 734 w, 726 m, 619 w. Crystallizations of powder product **1** from acetonitrile resulted either in crystals **3** or **4**. Crystals **3** were achieved in the presence of water if the solution was stored in an open beaker, while complex **4** crystallized in a closed system during a slow evaporation of solvent at reduced pressure.

Crystals **1** were obtained in the reaction of NiCl_2_**^.^**6H_2_O and Hhp in acetonitrile, described below in Section 3.2.4.

#### 3.2.2. Synthesis of [Ni(Hhp)_6_]Cl_2_, **2**

Crystals **2** grew during a slow evaporation of solvent at reduced pressure from a solution of powder product **4** in acetonitrile (see Section 3.2.4).

#### 3.2.3. Synthesis of [NiCl_2_(Hhp)(H_2_O)_2_]_2_·2(Hhp), **3**

Solvent (tetrahydrofuran, dichloromethane, or acetone, 20 mL) was added to Hhp (0.480, g, 5.05 mmol) and NiCl_2_**^.^**6H_2_O (0.300 g, 1.26 mmol). The yellow suspension was stirred at room temperature for seven days and then left to settle. The solution above the precipitate was filtered off. Crystals **3** grew out of the filtrate placed in an open beaker. The remaining precipitate was dried in a desiccator. An amount of 0.457 g of the complex **3** (51.0% yield) was gained. *Anal*. Calc. mass fractions of elements, *w*/%, for C_20_H_28_Cl_4_N_4_Ni_2_O_8_ (*M*_r_ = 711.6 g/mol): C, 33.75; H, 3.97; N, 7.87. Found: C, 33.74; H, 3.95; N, 7.80. IR (ATR cm^−1^) 3423 w, 3357 w, 3207 w, 3097 w, 3060 w, 2960 w, 2884 w, 2831 w, 1652 s (ν(CO)), 1614 m (ν(CO)), 1587 m (ν(CO)), 1543 m, 1468 m, 1454 w, 1428 m, 1400 m, 1382 w, 1265 w, 1234 m, 1219 m, 1166 w, 1154 w, 1094 w, 996 m, 919 w, 876 m, 852 m, 814 w, 772 vs, 725 vs, 619 w. A crystallization of powder product **3** from ethanol in an open beaker gained crystals **3**. A solution of **3** in acetonitrile was blue at the beginning, but yellow crystals **4** were achieved during the slow evaporation of solvent at reduced pressure for several days. 

#### 3.2.4. Synthesis of [Ni_2_Cl_4_(Hhp)_5_]·2MeCN, **4**

Solvent (acetonitrile, 20 mL) was added to Hhp (0.309 g, 3.25 mmol) and NiCl_2_**^.^**6H_2_O (0.309 g, 1.30 mmol). The suspension was stirred at room temperature for two days and then left to settle. The solution above the precipitate was filtered off and crystals **4** grew out of the green filtrate during a slow evaporation of solvent at reduced pressure. The remaining precipitate was dried in *vacuo*. An amount of 0.347 g of the complex **4** (65.3 % yield) was gained. *Anal*. Calc. mass fractions of elements, *w*/%, for C_29_H_31_Cl_4_N_7_Ni_2_O_5_ (*M*_r_ = 816.8 g/mol): C, 42.64; H, 3.83; N, 12.00. Found: 42.21; H, 3.85; N, 11.69. IR (ATR cm^−1^) 3218 w, 3079 w, 3059 w, 2936 w, 2810 w, 1640 s (ν(CO)), 1599 vs (ν(CO)), 1540 s, 1456 w, 1398m w, 1369 w, 1276 w, 1220 w, 1151 w, 1089 w, 998 m, 862 m, 773 vs, 721 m, 629 w. 

By increasing the metal-to-ligand molar ratio to 1:4 and prolonging the reaction time to 1 week, the crystals **1** were obtained in the yellow filtrate and complex **4** was precipitated, as confirmed by an IR spectrum. 

A crystallization of powder product **4** from acetonitrile in a closed system resulted in crystals **2**.

#### 3.2.5. Synthesis of [MnCl_2_(Hhp)_4_], **5**

Solvent (THF, 20 mL) and chlorotrimethylsilane (9.12 g, 84 mmol) were added to MnCl_2_**^.^**4H_2_O (0.336 g, 1.7 mmol) in the first step. The suspension was stirred at room temperature for four days and then left to settle. The solution above the precipitate was filtered off and discarded. The remaining precipitate was dried in vacuo and stored in nitrogen. Solvents, acetonitrile or methanol (10 mL), and Hhp were added to the precipitate in the second step. The molar ratios of manganese(II) to Hhp in the syntheses were 1:4 in acetonitrile and 1:6 in methanol. The resulting suspensions were stirred at room temperature for six days and then left to settle. The solutions above the precipitates were filtered off; crystals grew out of the filtrates in closed systems. Pure crystals **5** were gained in the system with a Mn/Hhp molar ratio of 1:4 and a mixture of colorless crystals **5** and brown Hhp crystals if the higher content of Hhp was used. The remaining precipitates were dried in vacuo and powder product **5** was gained. *Anal*. Calc. mass fractions of elements, *w*/%, for C_20_H_20_Cl_2_MnN_4_O_4_ (*M*_r_ = 506.2 g/mol): C, 47.45; H, 3.98; N, 11.07. Found: C, 47.02; H, 4.00; N, 10.95. IR (ATR cm^−1^) 3265 w, 3081 w, 3046 w, 2932 m, 1642 s (ν(CO)), 1598 s (ν(CO)), 1579 vs (ν(CO)), 1541 m, 1458 w, 1417 m, 1405 s, 1359 w, 1260 w, 1248 w, 1217 m, 1151 m, 1105 w, 1085 w, 1013 w, 992 m, 959 w, 907 m, 852 m, 825 m, 785 m, 771 vs, 725 m, 617 w. Crystals **5** were also achieved by a crystallization of powder product **5** from acetonitrile in a closed system. 

### 3.3. X-ray Structure Determinations

Each crystal was greased on a glass thread. The data were collected on an Agilent SuperNova Dual Source diffractometer with an Atlas detector, using either the graphite-monochromatized Mo-*Ka* radiation or the Cu-*Ka* radiation at 150 K. The data reduction and integration were performed with the software package CrysAlis PRO [88]. Corrections for the absorption (multi-scan) were made in all cases. All structures were solved by direct methods using either SIR–92 or SIR–2014 and refined against *F*^2^ on all data using a full-matrix least-squares procedure with SHELXL–2014 [89,90,91]. The positions of the NH hydrogen atoms in Hhp for all complexes and hydrogen atoms in water in complex **3** were unambiguously located from the residual electron density maps. Only the positions of the hydrogen atoms in water were refined using O–H distance restraints with *U*_iso_(H) = 1.5*U*_eq_(O). All the other hydrogen atoms were placed in geometrically calculated positions and refined using a riding model. Figures depicting the structures were prepared by ORTEP3 and Mercury [92,93].

## 4. Conclusions

The structural chemistry of nickel(II) and manganese(II) chloride complexes with pyridin-2-one (Hhp) is presented. The isolation of a single manganese and four nickel(II) complexes with Hhp under various conditions confirms the similar structural diversity of the nickel and copper complexes [36]. Hhp is coordinated in the manganese (**5**) and three nickel (**1**–**3**) complexes exclusively as a terminal O-donor ligand, only in the dinuclear complex (**4**) is terminal and bridging coordination of the ligand adopted. The metal ions are in an octahedral environment in all the prepared complexes (**1**–**5**). Six-numbered coordination geometry is accomplished merely by Hhp ligands in a homoleptic ion of **2** [Ni(Hhp)_6_]^2+^ or by four Hhp molecules and two chloride ions which are either in the *trans*, in [MCl_2_(Hhp)_4_] (**1** and **5**), or in the *cis* positions in [Ni_2_Cl_4_(Hhp)_5_]·2MeCN (**4**). Only one Hhp molecule together with three chloride ions in the *fac* and two water molecules in the *cis* positions render a coordination sphere of nickel in **3**. Solvate Hhp molecules in **3** are due to multiple hydrogen bonds also firmly held in the structure, as shown by thermal analyses. 

Copper(II) displays similar homoleptic complex ions [Cu(Hhp)_6_]^2+^ as nickel in **2** [34,54], but a different geometry in the molecular compounds due to the lower coordination numbers of the copper [36]. Nickel(II) and copper(II) ions afford two types of Hhp binding modes in dinuclear complexes, a terminal and a bridging one. Three Hhp molecules connect two nickel ions in **4** in a Ni_2_(*µ*-O)_3_ framework, but only two Hhp act as bridging ligands in [Cu(Hhp)_2_X_2_]_2_ due to a trigonal bipyramidal environment of copper [36]. Complex **4** is, to the best of our knowledge, the only example of a dinuclear nickel complex bridged by three neutral O-donor ligands.

To summarize, the four isolated octahedral nickel complexes with Hhp prove that various coordination numbers of metal ions as observed in similar copper compounds are not a prerequisite for structural diversity. Syntheses and characterizations of various Hhp complexes with zinc or some early *3d* metals are underway to gain a better understanding of the relationship of metal ions and the structural variety of these complexes.

## Figures and Tables

**Table 1:** Crystallographic data, data collection, and structure refinement data for the compounds **1**–**5**.

	1	2	3	4	5
Molecular formula	C_20_H_20_Cl_2_N_4_NiO_4_	C_30_H_30_Cl_2_N_6_NiO_6_	C_20_H_28_Cl_4_N_4_Ni_2_O_8_	C_29_H_31_Cl_4_N_7_Ni_2_O_5_	C_20_H_20_Cl_2_MnN_4_O_4_
Colour	yellow	yellow	yellow	yellow	colorless
For.mass (g/mol)	509.99	700.19	711.64	816.83	506.24
Cryst. syst.	monoclinic	monoclinic	triclinic	monoclinic	monoclinic
Radiation type	Mo *K*α	Mo *K*α	Cu *K*α	Mo *K*α	Mo *K*α
Space gr.	*P*2_1_/*n* (no. 14)	*P*2_1_/*n* (no. 14)	*P*$\bar{1}$ (no. 2)	*C*2/*c* (no. 15)	*P*2_1_/*n* (no. 14)
*a* (Å)	6.8918(6)	10.1083(10)	7.1191(4)	20.3325(6)	6.9071(3)
*b* (Å)	9.7906(7)	8.7836(6)	9.7675(5)	9.9564(3)	9.6873(4)
*c* (Å)	15.6618(15)	18.9985(15)	10.3811(4)	17.4575(5)	16.1391(8)
*α* (°)	90	90	68.572(4)	90	90
*β* (°)	90.316(5)	99.128(8)	86.606(4)	104.344(3)	90.020(4)
*γ* (°)	90	90	79.957(5)	90	90
*V* (Å^3^)	1056.8(2)	1665.5(2)	661.7(1)	3423.9(3)	1079.9(1)
*Z* (form.)	2	2	1	4	2
*D*_cal._ (g cm^−3^)	1.603	1.396	1.786	1.584	1.557
*μ* (mm^−1^)	1.207	0.793	5.958	1.461	0.894
Crystal size (mm)	0.16 × 0.16 × 0.12	0.4 × 0.4 × 0.5	0.17 × 0.17 × 0.12	0.4 × 0.3 × 0.2	0.16 × 0.14 × 0.12
*θ* Range (^o^)	3.0–27.5	3.1–27.5	4.6–74.6	3.1–27.5	3.2–27.5
Collected refl.	10229	9273	6162	16091	9807
Unique refl.	2409	3824	2694	3920	2476
*R*_int_	0.041	0.069	0.021	0.023	0.03
Observed refl.	2181	2600	2648	3575	2178
No. param.	143	205	184	218	142
*R*^a^ (*I* > 2.0 *σ*(*I*))	0.037	0.049	0.034	0.0327	0.027
*wR*_2_^b^	0.092	0.085	0.094	0.09	0.062
*S*	1.07	0.994	1.04	1.14	1.044
Max/min res. elec. d. (e Å^−3^)	−0.54, 0.53	−0.61, 0.53	−0.76, 0.48	−0.85, 0.74	−0.23, 0.28

^a^*R* = Σ(|*F*_o_| − |*F*_c_|)/Σ|*F*_o_|, ^b^ *wR*_2_ = (Σ[*w*(*F*_o_^2^ − *F*_c_^2^)^2^]/Σ(*wF*_o_^2^)^2^)^1/2^.

**Table 2:** Selected geometric parameters (Å, °) in [NiCl_2_(Hhp)_4_] (**1**), [MnCl_2_(Hhp)_4_] (**5**) and [Ni(Hhp)_6_]Cl_2_ (**2**).

	**1**	**5**		**1**	**5**
M1–O1	2.036(2)	2.1401(11)	O1–M1–O2	89.59(7)	87.50(4)
M1–O2	2.078(2)	2.1764(11)	O1–M1–O2 ^i^	90.41(7)	92.50(4)
M1–Cl1	2.4539(2)	2.5762(4)	O1–M1–Cl1	86.79(6)	87.78(3)
O1–C11	1.254(3)	1.2562(18)	O2–M1–Cl1	91.69(6)	91.52(3)
O2–C21	1.266(3)	1.2626(19)	O1–M1–Cl1 ^i^	93.21(6)	92.22(3)
			O2–M1–Cl1 ^i^	88.31(6)	88.48(3)
			M1–O1–C11	133.23(19)	136.35(10)
			M1–O2–C21	130.64(18)	131.91(9)
Symmetry codes in **1** and **5**: (i) 1 − *x*, −*y*, 1 − *z*.					
**2**					
Ni1–O1	2.0387(19)		O1–Ni1–O2	88.27(7)	
Ni1–O2	2.0489(19)		O1–Ni1–O3	89.19(7)	
Ni1–O3	2.0530(19)		O2–Ni1–O3	93.90(7)	
O1–C11	1.256(3)		O1–Ni1–O1 ^i^	180.00	
O2–C21	1.273(4)		O1–Ni1–O2 ^i^	91.73(7)	
O3–C31	1.274(3)		O1–Ni1–O3 ^i^	90.81(7)	
			O2–Ni1–O3 ^i^	86.10(7)	
			Ni1–O1–C11	128.64(19)	
			Ni1–O2–C21	128.41(17)	
			Ni1–O3–C31	127.80(17)	
Symmetry codes in 2: (i) −*x*, −*y*, −*z*.					

**Table 3:** Selected geometric parameters (Å, °) in **3**, [NiCl_2_(Hhp)(H_2_O)_2_]_2_·2Hhp.

Ni1–Cl1	2.4111(6)	Cl1–Ni1–Cl2	90.19(2)
Ni1–Cl2	2.3975(6)	Cl1–Ni1–O1	174.75(4)
Ni1–Cl2 ^i^	2.4010(6)	Cl1–Ni1–O2	93.02(4)
Ni1–O1	2.1503(15)	Cl1–Ni1–O10	96.53(5)
Ni1–O2	2.0639(15)	Cl1–Ni1–Cl2 ^i^	94.15(2)
Ni1–O10	2.0293(15)	Cl2–Ni1–O1	84.56(4)
O10–C10	1.265(3)	Cl2–Ni1–O2	174.77(5)
		Cl2–Ni1–O10	97.46(4)
		Cl2–Ni1–Cl2 ^i^	89.29(2)
		O1–Ni1–O2	92.22(6)
Ni1^…^Ni1 ^i^	3.4141(5)	O1–Ni1–O10	84.13(6)
		Cl2 ^i^–Ni1–O1	85.86(4)
		O2–Ni1–O10	86.28(6)
		Cl2 ^i^–Ni1–O2	86.37(5)
		Cl2 ^i^–Ni1–O10	167.33(5)
		Ni1–Cl2–Ni1 ^i^	90.71(2)

Symmetry code: (i) 2 − *x*, −*y*, −*z*.

**Table 4:** Selected geometric parameters (Å, °) in **4**, [Ni_2_Cl_4_(Hhp)_5_]·2MeCN.

Ni1–Cl1	2.3690(7)	Cl1–Ni1–Cl2	93.64(2)
Ni1–Cl2	2.3628(6)	Cl1–Ni1–O1	96.86(5)
Ni1–O1	2.0219(16)	Cl1–Ni1–O2	94.34(5)
Ni1–O2	2.0882(16)	Cl1–Ni1–O3	96.52(3)
Ni1–O2 ^i^	2.1078(17)	Cl1–Ni1–O2 ^i^	167.82(5)
Ni1–O3	2.0892(14)	Cl2–Ni1–O1	94.96(5)
O1–C11	1.263(3)	Cl2–Ni1–O2	96.25(5)
O2–C21	1.271(3)	Cl2–Ni1–O3	166.97(4)
O3–C31	1.268(4)	Cl2–Ni1–O2 ^i^	94.21(5)
		O1–Ni1–O2	163.60(7)
Ni1^…^Ni1 ^i^	2.9802(5)	O1–Ni1–O3	91.91(5)
		O1–Ni1–O2^i^	91.72(7)
		O2–Ni1–O3	74.91(6)
		O2–Ni1–O2 ^i^	74.50(6)
		Ni1–O1–C11	128.76(16)
		Ni1–O2–C21	134.85(15)
		Ni1–O3–C31	134.49(4)
		Ni1–O2–Ni1 ^i^	90.53(7)
		Ni1–O3–Ni1^i^	91.01(8)

Symmetry code: (i) 2 − *x*, *y*, 1.5 − *z*.

## Supplementary Materials

The following are available online. Table S1. Hydrogen-bond geometry (Å, °) in [NiCl_2_(Hhp)_4_] (**1**), [Ni(Hhp)_6_]Cl_2_ (**2**), [MnCl_2_(Hhp)_4_] (**5**). Table S2. Hydrogen-bond geometry (Å, °) in **3**, [NiCl_2_(Hhp)(H_2_O)_2_]_2_·2Hhp. Table S3. Hydrogen-bond geometry (Å, °) in **4**, [Ni_2_Cl_4_(Hhp)_5_]·2MeCN.
