# Supplementary material for: Structural Diversity of Nickel and Manganese Chloride Complexes with Pyridin-2-One
Source: Molecules. 2020 Feb 14;25(4):846. doi: 10.3390/molecules25040846 (PMC7070377; doi:10.3390/molecules25040846)
Supplement: Supplementary file 1 [file molecules-25-00846-s001.pdf]

Table S1. Hydrogen-bond geometry (Å, °) in $[NiCl_2(Hhp)_4]$ (**1**), $[Ni(Hhp)_6]Cl_2$ (**2**), $[MnCl_2(Hhp)_4]$ (**5**).

D–**H**···**A**	***D***–**H**	**H**···**A**	***D***···**A**	***D***–**H**···**A**
		**1**		
N1–H1···O2	0.8800	2.0000	2.968(3)	146.00
N2–H2···Cl1^{i}	0.8800	2.0000	3.076(2)	175.00
		**2**		
N1–H1···O2	0.8600	2.5200	2.949(3)	112.00
N1–H1···O3^{i}	0.8600	2.0800	2.821(3)	143.00
N2–H2···Cl1	0.8600	2.2900	3.150(3)	174.00
N3–H3···Cl1^{i}	0.8600	2.3000	3.142(3)	167.00
		**5**		
N1–H1···O2	0.8600	2.4300	3.176(2)	145.00
N2–H2···Cl1^{i}	0.8600	2.2300	3.090(1)	175.00

Symmetry code in **1**: (i) $-1 + x, y, z$; in **2**: (i) $-x, -y, -z$; in **5**: (i) $1 + x, y, -z$.

Table S2. Hydrogen-bond geometry (Å, °) in **3**, $[NiCl_2(Hhp)(H_2O)_2]_2{\cdot}2Hhp$.

D–**H**···**A**	***D***–**H**	**H**···**A**	***D***···**A**	***D***–**H**···**A**
O1–H1A···O20	0.89(3)	2.04(3)	2.911(2)	167(3)
O2–H2A···Cl1^{i}	0.85(3)	2.35(3)	3.1810(16)	170(3)
O2–H2B···O20^{ii}	0.86(3)	1.90(3)	2.755(2)	171(3)
N10–H10···O20^{iii}	0.8800	1.9200	2.793(2)	171.00
C21–H21···O10^{iv}	0.9500	2.2500	3.160(3)	159.00

Symmetry code: (i)$1- x, -y, -z$; (ii) $-1 + x, y, z$; (iii) $2 - x, 1 - y, -z$; (iv) $2 - x, -y, 1 - z$.

Table S3. Hydrogen-bond geometry (Å, °) in **4**, $[Ni_2Cl_4(Hhp)_5]{\cdot}2MeCN$.

D–**H**···**A**	***D***–**H**	**H**···**A**	***D***···**A**	***D***–**H**···**A**
N1–H1···Cl1	0.8800	2.3600	3.178(2)	154.00
N1–H2···Cl2	0.8800	2.3300	3.128(2)	150.00
